# ﻿The effect of formaldehyde solution in pitfall traps on the probability of catching woodlice (Isopoda, Oniscidea)

**DOI:** 10.3897/zookeys.1225.123647

**Published:** 2025-02-05

**Authors:** Ivan Hadrián Tuf, Lucie Novotná, Pavel Fryčka

**Affiliations:** 1 Department of Ecology and Environmental Sciences, Faculty of Science, Palacký University Olomouc, Šlechtitelů 27, Olomouc, CZ-77900, Czech Republic Palacký University Olomouc Olomouc Czech Republic

**Keywords:** Barber traps, epigeic invertebrates, epigeon, model species, terrestrial isopods, trapability

## Abstract

Pitfall traps containing a fixative solution are commonly used by ecologists to study ground-dwelling invertebrates. The effect of the solution on the animals being caught is a frequent topic of studies. Our study compares the effect of formaldehyde solution, water, and the random probability of catch simulated by using dry traps. Ninety pitfall traps were placed in a floodplain forest ground: one-third used 4% formaldehyde solution as a fixative, one-third used water, and one-third was left without any liquid to simulate the random probability of a catch. A layer of dry wood chips was used in the dry traps to reduce predation between the caught animals. The traps were placed in the field between April and May 2022 and emptied twice a week. Both the numbers of animals and the species caught were found to be significantly affected by the fixative solution in use. Significantly more woodlice were caught in traps filled with water; these traps also attracted significantly more *Porcelliumconspersum* and *Trachelipusrathkii* compared to the dry traps. Average day temperature and the order the traps were checked (i.e. date) had an influence on the animals caught as well. Additional laboratory experiments with *Porcellioscaber* confirmed that terrestrial isopods avoid formaldehyde pitfall traps more than those with water.

## ﻿Introduction

Although the use of pitfall traps was first mentioned more than a hundred years ago, it was not described in detail until the publication by Herbert Spencer Barber, who used traps to study cave invertebrates (Barber 1931). Pitfall traps, now also called Barber´s traps, are considered to be the most widely used method for studying epigeic invertebrates (Bater 1996). This method is popular not only because of its affordability and ease of construction (Hohbein and Conway 2018) but also its effectiveness (Topping and Sunderland 1992).

The basic form of a pitfall trap is an open container buried into the ground, with the edge of the trap exposed on the ground level. The original traps constructed by Barber used glass tubes with rotting meat as bait and ethanol as a fixative solution. Since then, a large number of studies and reviews tried to find the best universal design for pitfall traps (e.g., Adis 1979; Knapp et al. 2016).

Various forms of bait have been tested, from rotting meat or smelly cheese to rotting fruit (Silva et al. 2012), cattle dung or blood (Gatty and Grández 2020) or human faeces (Filgueiras et al. 2009). Bait can be used to massively increase the number of caught invertebrates, especially in the case of predatory and scavenging species. Bait in liquid form, such as beer (Santalla et al. 2002) or wine (Baini et al. 2016), can also serve as a fixative solution. It is important to note that the fixative solution (Gerlach et al. 2009) or trapped animals can also act as unintentional bait.

Fixative solution can be used to quickly kill and prevent decomposition of the captured animals (Knapp and Růžička 2012). Pitfall traps without preservative solution are occasionally used to catch epigeon for breeding, feeding pet reptiles or amphibians, experiments or educational purposes. In that case, it is recommended to fill them partly with dry leaves, crumpled wet newspaper, moss or wire mesh to reduce predation between the caught specimens (Skuhravý 1957; Hatten et al. 2007).

One of the disadvantages of using a fixative solution is its possible effect as an attractant or repellent for different species (Adis 1979). Many scientists have evaluated the effects of water, salt solution, ethanol, ethylene glycol, propylene glycol, formaldehyde, paraffin, vinegar, and others (Knapp 2007), primarily focusing on maximizing the number of caught animals. In contrast, our study aims to understand how the fixative solution (formaldehyde or water) affects the catch size of terrestrial isopods in general. The behaviour of a model species of woodlouse was tested in a natural as well as a controlled laboratory environment.

## ﻿Material and methods

### ﻿Field experiment

The site chosen for the experiment was the floodplain forest in the Litovelské Pomoraví Protected Landscape Area near Olomouc, Czech Republic (49°39'11.1"N, 17°12'42.3"E, total size 179 ha). The tree floor in the selected part of the forest was dominated by hornbeam (*Carpinusbetulus*), linden (*Tiliaplatyphyllos*) and oak (*Quercusrobur*) with an admixture of maples (*Acercampestre*, *Acerpseudoplatanus*). Mean temperatures during the study period (April and May 2022) were comparable to the long-term average, with minimum daily temperatures ranging between -3.1 and 16.4 °C and maximum daily temperatures between 3.2 and 28.1 °C. Precipitation was strongly above the long-term average (higher by c. 33%).

The sampling of ground-dwelling invertebrates was carried out continuously from April 2 to May 28 using 90 pitfall traps. Each trap was made from a glass jar with an inserted plastic cup of inner diameter 6.5 cm and depth 10.5 cm and covered by a metal hood 2 cm above ground. The traps were placed in nine lines of ten at regular 12 m intervals between each. Thirty pitfall traps contained a 4% formaldehyde fixative solution, 30 traps were filled with plain water (without added detergent) and 30 were left dry. The distribution of traps by their content was not random, but regular by lines. Dry traps were partially filled by dry wood shavings to prevent predation between caught animals. The traps were collected each Tuesday and Friday for a period of 9 weeks (i.e. 17 inspections), and the collected material was stored in a freezer box. Subsequently, the captured terrestrial isopods were identified.

### ﻿Laboratory experiment

A design similar to the experiment by Gerlach et al. (2009) was used to monitor the animal behaviour. A hole was cut in the middle of the bottom of a 17 × 17 × 11 cm plastic container to insert a glass jar (height 15 cm, neck width approximately 7.7 cm, volume 0.7 L) so that the top of the jar protruded approximately 4 cm inside the container. The container was then filled with a layer of gypsum up to the neck of the jar. Gypsum was used to retain moisture and was covered by a thin layer of soil. The portion of the glass jar below the container was then wrapped in black foil to prevent light from coming into the trap. Shelters (rocks and brick fragments) were placed in each of the four corners of the apparatus, along with pieces of decaying herbs.

**Figure 1.**
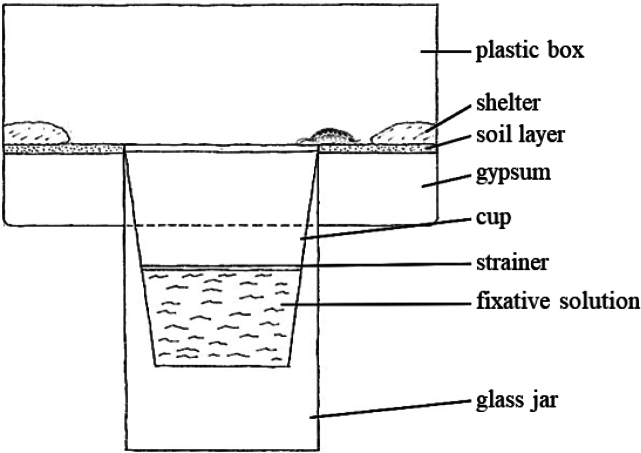
Laboratory apparatus imitating pitfall trap with surrounding soil surface.

A plastic cup with a neck diameter of approximately 7.7 cm and a height of 11 cm was placed into the jar. A plastic strainer was fixed c. 5 cm above the bottom of the cup to prevent animals from falling into the fixative solution poured just below the strainer. In total, two identical apparatuses were assembled. One with a trap containing water and one with a trap containing a 4% formaldehyde solution (Fig. 1). A Genius FaceCam 311 webcam, connected to a laptop, was mounted above the trap to record animal behaviour.

*Porcellioscaber* Latreille, 1804, easily available in sufficient numbers, was chosen as a model species for laboratory experiments. A group of 10 adult individuals, collected in parks in Olomouc, Czech Republic, was placed in the trap container each day at 17:00 h. The trap was covered for 15 minutes to prevent immediate capture of the animals. Recording ran until 14:00 the following day. Because the cameras were unable to record during the twilight or darkness, each container was illuminated with a 40W red bulb throughout the experiment; isopods are at least sensitive to longer wavelengths (Hariyama et al. 2001). The experiment was run three times with water and three times with formaldehyde at room temperature (c. 20 °C) and with a natural light regime. A new group of animals was used for each recording.

### ﻿Statistical analyses

#### ﻿Field experiment

Mean catches as a function of fixation solution were compared using univariate ANOVAs, and Tukey’s tests were used to test the significance of differences between pairs of trap types. Data analysis was performed using CANOCO for Windows 5.0. At the beginning of the analysis, it was necessary to determine the dependent (species data) and independent (environmental data) variables. Species data represented the number of trapped individuals for each species. The environmental data represented trap type (categorical variables “water”, “formaldehyde” and “dry”), average daily temperature one (t-1), two (t-2), and three (t-3) days prior to trap inspection, average temperature for those three days (“*t* mean”), and number of collecting occasion (“sample”).

The length of the gradient in the species data (3.58) allowed the use of a direct linear gradient analysis (RDA) that plots multivariate relationships between species and environmental data. To express the relationships between temperature and the number of individuals of captured species, generalized linear models (GLMs) were used. Statistical significance and model power were tested using a Monte Carlo permutation test (499 repetitions). All the above diagrams generated by the linear method were created in CanoDraw for Windows.

#### ﻿Laboratory experiment

The activity of the animals during the experiment was read from the recordings obtained. The movement of an individual in a 2 cm band around the edge of the trap was taken as “one behavioural act”. The total length of the recording was divided into 2-hour segments to which activity counts were assigned. Activity values obtained during the first two hours of observation (i.e., 17:00 to 19:00) were omitted due to the strikingly high movement of animals associated with their placement in a new environment. Animal behaviour observed was classified (cf., Gerlach et al. 2009) into these categories:

1. “nearby trap” activity - passing through the observed area without contact with the trap and without a change of direction or passing through the observed area without contact with the trap with a change of direction;
2. “in contact” activity - changing of direction after contact with the trap or going over the edge of the trap with part of the body and backing out;
3. “watching inside” activity - going over the edge with more than half of the body and backing out;
4. “trapped” activity - getting caught in the trap.

The number of occurrences for each category was recorded, with each next category including the previous ones, i.e., each “trapped” individual was “watching inside” before, which was the result of “in contact”, etc. The mean values of the above categories were calculated from the three repetitions with the given fixative solution. The “Rate of Self-Rescue” (Gerlach et al. 2009), i.e., the percentage of animals that were able to climb out of the trap after overstepping its edge by more than half of their body to the number of animals that overstepped the trap edge by more than half of their body that was trapped, was used to represent the ability of a species to rescue itself from falling into the trap. Line graphs were used to graphically represent the values in the categories. The statistical significance of differences between these categories for water traps and formaldehyde traps within species was determined using a goodness-of-fit test.

## ﻿Results

### ﻿Field experiment

During the two-month study in the Litovelské Pomoraví Protected Landscape Area, a total of 2910 terrestrial isopods of 8 species were captured using 90 pitfall traps. On average, 26.9 ± 15.9 individuals were caught in a single dry trap, 25.4 ± 15.6 individuals were caught in a trap with formaldehyde, but significantly more individuals were caught in a trap with water (44.7 ± 23.0 ind.; *F* = 10.17, *p* < 0.001).

**Figure 2.**
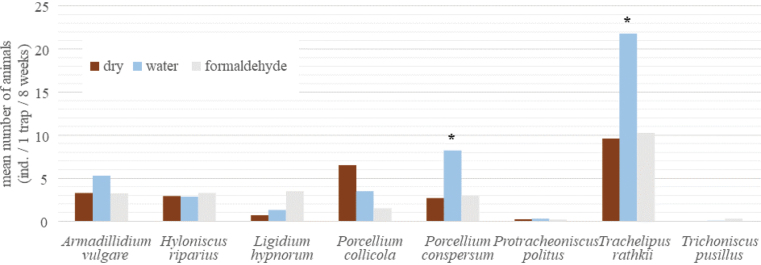
Mean numbers of trapped terrestrial isopods in traps differing in fixative solution during spring months in floodplain forest. Asterisk marks significantly different caught of particular species suggesting relative attractivity of marked solution in comparison with other treatments.

It is evident (Fig. 2), that using formaldehyde fixative solution did not significantly affect trap efficiency compared to the dry pitfall traps. The most abundant trapped species was *Trachelipusrathkii* (43% of the catch, Fig. 2). This species was significantly more likely to be caught in water traps than in dry traps or formaldehyde traps, similar to *Porcelliumconspersum*. The other species did not show significant differences in catch size for a particular type of trap, although *Armadillidiumvulgare* was insignificantly more numerous in traps with water and *Ligidiumhypnorum* was more numerous in traps with formaldehyde solution.

**Figure 3.**
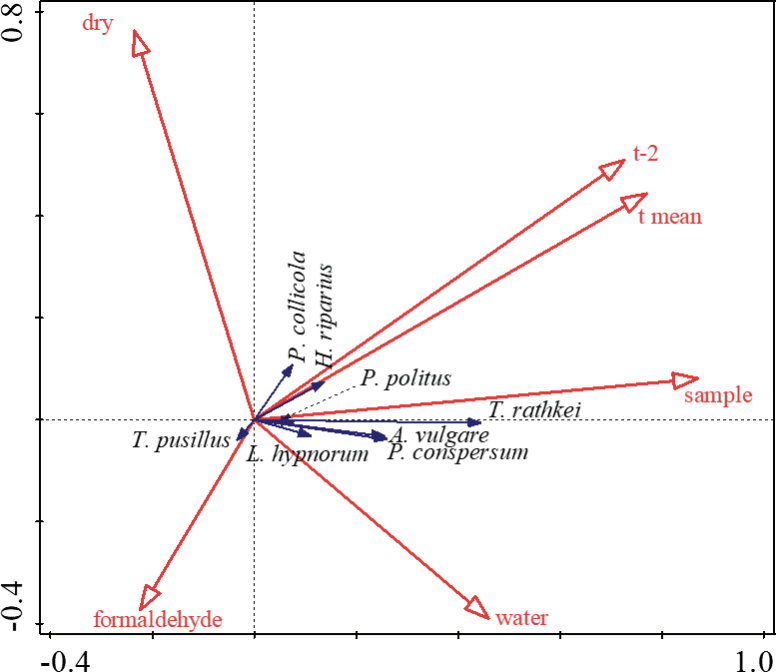
RDA biplot illustrating the effect of environmental factors (red) on trapping probability of terrestrial isopods. Only significant factors are illustrated: “*t mean*” – mean temperature three days before trap inspection, *t-2* – mean temperature during the second day before trap inspection, “*sample*” – order of trap inspection, “*formaldehyde*/*water*/*dry*” – types of fixative solution in pitfall traps.

A direct linear gradient analysis (RDA) looked at the effect of the trap fill (dry/water/formaldehyde), average daily temperature between trap inspections, and the effect of season (expressed as number of trapping occasion) on the abundance of trapped woodlouse (Fig. 3). The first axis of the model explains 8.2% of the variability (*pseudo-F* = 24.0; *p* = 0.002), and the model is significant (*pseudo-F* = 27.2; *p* = 0.002). Simple term effect was significant for all tested factors (*p* < 0.05) but conditional term effect highlighted changes associated with season (“sample”, explains 6.24%, *pseudo-F* = 108, *p* = 0.002), temperature 2 days before trap inspection (*t-2*, explains 0.53%, *pseudo-F* = 9.3, *p* = 0.002) and fixative solution – against “formaldehyde”, “water” explains 1.9% (*pseudo-F* = 33.5, *p* = 0.002) and “dry” traps explain 0.27% of variability in species abundances (*pseudo-F* = 4.8, *p* = 0.002). The last valuable environmental factor for RDA was mean temperature (“*t* mean”, explains 0.18%, *pseudo-F* = 3.1, *p* = 0.028).

**Figure 4.**
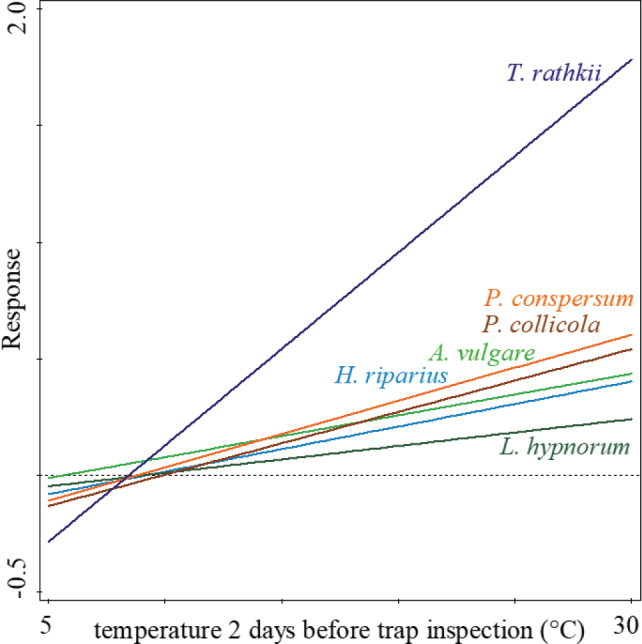
Generalised linear model for the effect of daytime mean temperature two days before trap inspection on terrestrial isopods´ probability to be trapped. Only species with significant predictions are illustrated (see Table 1).

Fitted generalized linear models (GLM) revealed a significant positive effect between temperature two days before trap inspection and the number of trapped animals of all terrestrial isopod species, except two less abundant species (Table 1, Fig. 4).

**Table 1.** Summary of fitted Generalised Linear Model of mean day temperature two days before trap inspection predicting number of trapped terrestrial isopods. R-squared represents the proportion of the deviance explained by the model, F value is a value on the F distribution (power of result) and the p-value compares the fitted model to a null model.

Response	R2[%]	F	p
*Trachelipusrathkii* (Brandt, 1833)	10.4	188.4	<0.00001
*Hyloniscusriparius* (C. Koch, 1381)	3.1	51.1	<0.00001
*Porcelliumconspersum* (C. Koch, 1841)	2.7	45.3	<0.00001
*Armadillidiumvulgare* (Latreille, 1804)	1.8	29.4	<0.00001
*Ligidiumhypnorum* (Cuvier, 1792)	1.1	18.6	0.00002
*Porcelliumcollicola* (Verhoeff, 1907)	1.1	17.4	0.00003
*Protracheoniscuspolitus* (C. Koch, 1841)	0.2	3.8	0.05084
*Trichoniscuspusillus*Brandt, 1833	0.0	0.5	0.50501

### ﻿Laboratory experiment

**Figure 5.**
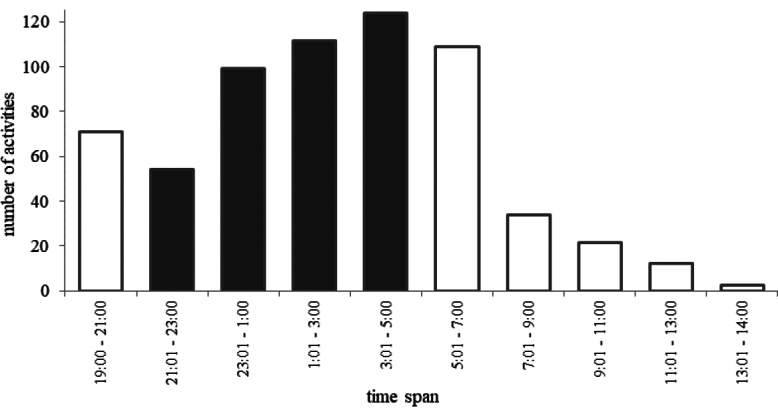
Pattern of activity of *Porcellioscaber* in experimental apparatuses with pitfall trap, expressed as the number of behavioural acts counted during particular two-hour intervals. Dark columns correspond to night hours.

**Figure 6.**
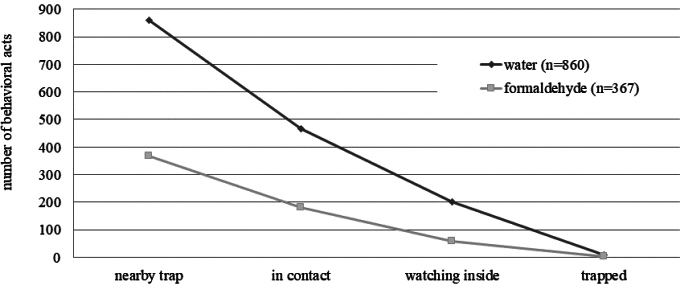
Behavioural acts of *Porcellioscaber* counted in experimental apparatuses with pitfall traps filled with water and formaldehyde, respectively. For explanation of categories see Material and methods.

*Porcellioscaber* activity was highest between 23:00 and 7:00 (Fig. 5). Higher activity was recorded for *P.scaber* in the water-filled trap apparatus; this difference in overall “nearby trap” count was statistically significant (*χ^2^* = 198.88, *p* < 0.001, Fig. 6). When walking near the trap (at a distance of 2 cm or less), approximately half of the behavioural acts represented “contact” with the trap (54% for the water-filled trap and 50% for the formaldehyde-filled trap, cf., Fig. 6) the difference between the formaldehyde and water trap also being significant in this case (*χ^2^* = 124.47, *p* < 0.001, Fig. 6). Only 23% and 16% of isopods being “nearby trap” (the water- and formaldehyde-filled traps, respectively) were “watching inside” the trap, i.e., inserting more than half of the body beyond the trap edge. This difference was also statistically significant (*χ^2^* = 198.88, *p* < 0.001, Fig. 6). Regardless of the type of the fixative solution, falling into the trap was very rare, with only a few “trapped” individuals, and there was no difference between the two groups (*χ^2^* = 1.33, *p* = 0.85). The “Rate of Self-Rescue” of *P.scaber* in the water trap apparatus was 67% (only 8 “trapped” individuals out of 24 that overstepped more than half of their body behind the edge of the trap), while in the formaldehyde trap, the rate was 78% (4 individuals of 18).

## ﻿Discussion

Numerous modifications of pitfall traps used by field ecologists make it impossible to easily compare data between different studies. In this study, we tested the effect of formaldehyde and plain water on the probability of trapping terrestrial isopods, using the dry pitfall trap results as a baseline probability of capture. The dry traps were used as the baseline because we assume they do not contain anything that could attract the animals. There still could be one factor in play, the tendency of woodlice to actively avoid falling into the trap (see laboratory experiment below), but this would affect all the pitfall traps regardless of the fixative solution used.

We believe that water traps were more attractive to terrestrial isopods, as they need to visit shelters with high atmospheric humidity to compensate for their own loss of water (Wright and Machin 1990). Studies have shown that the most preferred level of humidity for terrestrial isopods is between 77 and 94% (Snyder 1959). Woodlice, in general, are hygrophilous (Hornung 2011), and *T.rathkii* and *P.conspersum* have similarly low desiccation resistance (around 25 hours) (Dias et al. 2012) and, because of that, have a similar need to seek humidity. We found these two dominant species significantly more likely to be captured in water traps. We also assume that the ambient humidity under the metal hoods above the water traps was higher, although we did not measure this factor.

Predation may have also influenced the results for traps without killing fixative solutions (Bouget 2001). Despite frequent inspections of traps and lining their bottom with wood shavings, remains of body parts of smaller species (not only woodlice) were found. We sought to minimize this effect by collecting the trapped animals every three to four days. It seems that the random chance of capture by dry traps was somewhat underestimated.

Formaldehyde, as the fixative solution used, acts as an attractant for ground beetles (Luff 1975) but as a repellent to the harvestmen (Pekár 2002), where a decrease in the number of individuals caught can be observed with the increasing concentration of formaldehyde solution. On the contrary, spiders were not significantly affected by the changing concentration (Pekár 2002). Although Gerlach et al. (2009), among others, did not observe any attraction or repulsion effect of water for ground-dwelling arthropods under laboratory conditions, significantly more terrestrial isopods were caught in water traps in this study. Nevertheless, the number of trapped isopods in formaldehyde traps was similar to the number of isopods in dry traps, which gave random (i.e., not affected by attraction nor repulsion effect) trapping probability value.

In our laboratory experiment, formaldehyde was confirmed to have a repellent effect on *P.scaber*. The specimens near the formaldehyde trap were less active and had a higher “Rate of Self-Rescue” compared to the traps filled with water. Similar results were found by Gerlach et al. (2009) for *Armadillidiumopacum* (Koch, 1844) and *Oniscusasellus* Linnaeus, 1758.

In addition to the effect of the fixative solution, the effect of average daily temperature between trap inspections on the trap catch size was also observed. The catch size in pitfall traps increased with increasing temperature. Although high temperatures were observed to decrease the activity of woodlice in a laboratory setting (Ďurajková et al. 2022), the temperature in the forest was much lower than in a laboratory and its increase may have led to an increased woodlice activity. A similar relationship between soil temperature (and humidity) and the activity of terrestrial isopods during spring was observed by Hornung et al. (2015). Since formaldehyde is volatile, it likely evaporated more as the temperature increased during the time of our study, which may have altered the strength of the fixative solution effect. At the same time, ground-dwelling species bound to moist environments may have searched for water as the temperature increased and thus fell into the pitfall traps with clear water more often. Rendoš (2012) found that terrestrial isopods significantly avoided formaldehyde traps even in subterranean environments where sufficient moisture is expected throughout the year.

It does not seem necessary to change a proven fixative solution (formaldehyde) in further ecological field studies, apart from its toxicity. It can be assumed that its use in traps will continue to provide valuable results. However, it should be kept in mind that the data obtained by this method may be skewed, especially when studying a community structure and dominant species in an area – some species are more sensitive to formaldehyde and may actively avoid it. Obtained community characteristics are comparable with another similarly skewed, but such comparison is based on unmonitored changes in the abundances of sensitive species.

## ﻿Additional information

### Author ORCIDs

Ivan Hadrián Tuf https://orcid.org/0000-0003-0250-0482

### Data availability

All of the data that support the findings of this study are available in the main text.
